# Double optic capture: a historical review

**DOI:** 10.3389/fopht.2025.1700902

**Published:** 2025-12-09

**Authors:** Brian M. DeBroff

**Affiliations:** Department of Ophthalmology and Visual Science, Yale University School of Medicine, New Haven, CT, United States

**Keywords:** double optic capture, posterior capsulorhexis, capsular bag fusion, posterior capsulorhexis with optic capture, pediatric cataract surgery, posterior optic buttonholing

## Abstract

The surgical technique of double optic capture involves capturing the optic of an intraocular lens implant through both an anterior and posterior capsular opening as a method to reduce secondary capsule opacity and membranes, while ensuring intraocular lens (IOL) centration. The techniques of performing this procedure is described as well as newer surgical modifications of the technique for both pediatric and adult cataract surgery. Future directions involving its use in routine adult cataract surgery as a method to prevent posterior capsule opacity are discussed.

## Introduction

**Figure 1:**
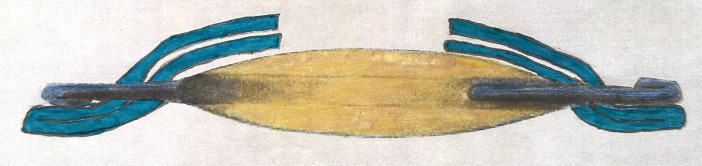
Schematic diagram depicting an IOL in the double optic captured position: IOL haptics in the sulcus and IOL optic captured through both the anterior and posterior capsulotomy openings.

Double optic capture, which involves double capturing an intraocular lens implant through both an anterior and posterior capsular opening, was first described in 2008 by DeBroff and Nihalani in 2008 as a method to reduce visual axis opacification while ensuring IOL centration in pediatric cataract surgery (1). DeBroff and Nihalani presented 16 eyes with a mean follow-up of 1.7 years that maintained IOL centration, had minimal anterior chamber reaction, and had no evidence of visual axis opacification (1). The technique of double optic capture involves performing a continuous curvilinear capsulorhexis of the anterior capsule, removal of the cataract, performing a posterior capsulotomy opening (either using a vitrectomy handpiece to match the size of the anterior capsulorhexis or performing a primary posterior capsulorhexis), anterior vitrectomy if needed, and placement of IOL haptics in the ciliary sulcus, followed by capture of the IOL optic through both the anterior and posterior capsular openings (**Figure 1**) (1–4). Because of the contraindication for a one-piece IOL in the sulcus, it recommended that ideal IOL implant for double optic capture should be a three-piece IOL. The IOL diameter can vary as long as the capsular opening is ideally at least 1 mm smaller than the IOL optic to allow for optimal and secure capturing of the IOL optic. Double optic capture allows for fusion of the anterior and posterior leaflets of the capsular bag 360°, which effectively seals the capsular bag. This fusion of the capsule leaflets reduces the possibility of lens epithelial cell migration and thus reduces the incidence of secondary membrane formation while securing in the IOL position in a locked position, preventing decentration. Finally, due to the secure and stable fixation of the IOL, there is minimal haptic rub of uveal tissues and a low incidence of persistent anterior chamber inflammation postoperatively. Onol et al. in 2000 described using the double optic capture technique in combination with pars plana lensectomy in pediatric cataracts (5) and subsequently published a series with long-term follow-up demonstrating IOL stability and clarity of the visual axis in 16 eyes (6). Other surgeons have described excellent results utilizing the double optic capture technique as a preferred technique to maintain IOL centration and prevent secondary membrane formation (7).

## Surgical technique

DeBroff and Gimbel in 2004 reported that DOC can be achieved by performing both an anterior capsulorhexis and a posterior capsulorhexis and placing the IOL optic through both the anterior and posterior capsulorhexis openings (8). Because performing a posterior capsulorhexis can be technically challenging, especially to create a circular opening approximately 1 mm smaller than the IOL optic diameter in an elastic pediatric capsule, femtosecond laser may be a future option to perform not only the anterior capsular opening but also the posterior capsular opening (2, 9–12). Several companies are exploring the option of enabling posterior capsulotomy opening on their commercially available femtosecond laser platforms. Surgeons who are technically adept with consistently performing a posterior capsulorhexis of a diameter 1 mm or more smaller than the optic diameter may consider the technique of posterior capsulorhexis with optic capture (PCOC), which involves in-the-bag IOL placement with optic capture through the posterior capsulorhexis (13, 14). The downside to PCOC (as compared with double optic capture) is that the fusion of the anterior and posterior capsules are not complete at 360°. There is lack of fusion at the optic haptic junctions and thus a discontinuous fusion as compared with double optic capture (15, 16). Another potential downside of performing posterior capsulorhexis is the possibility of peripheral extension of a posterior capsulotomy which often precludes the placement of the haptics in the capsular bag. When performing double optic capture, the author only recommends proceeding with a posterior capsulotomy opening if the anterior capsulotomy opening is completely intact. In the event of an extension of the posterior capsular opening, secure IOL positioning could still be achieved with rhexis IOL fixation (three-piece IOL haptics in the sulcus with capturing the IOL optic through the anterior capsulorhexis opening), which was first described by Neuhann and Neuhann in 1991 (8).

Performing a posterior capsulorhexis, however, with either double optic capture or posterior capsulorhexis with optic capture has the benefit of potential elimination of the need to perform an anterior vitrectomy at the time of surgery (17, 18). By avoiding an anterior vitrectomy, the surgeon can reduce inflammatory sequalae and decrease the incidence of cystoid macular edema, especially in adult patients (19). In cases where removal of posterior lenticular plaques or vascular stalk remnants are needed, such as in cases of PHPV, a vitrectomy is required. DeBroff described a case of surgical treatment of a unilateral congenital cataract with PHPV using double optic capture that after 20 years achieved a clear visual axis and best corrected visual acuity of 20/20-2 (20). Also, Dr. Howard Gimbel presented a case of double optic capture utilizing posterior capsulorhexis without anterior vitrectomy to successfully treat a congenital pediatric cataract associated with Down’s syndrome (21). In 2014, DeBroff reported that with double optic capture, even if the anterior capsulorhexis is larger than the IOL optic, the posterior capsulotomy size can be adjusted smaller than the IOL optic to enable effective and secure capture (2). Also, IOL optic centration has been shown to be maintained even in cases of eccentric anterior capsulorhexis (22).

Finally, it has been demonstrated that it is possible to perform IOL exchange with recapturing of a new IOL many years after double optic capture if this is required (20).

## Surgical applications of the procedure

A major complication of traditional IOL implantation in the pediatric population is the frequent complication of posterior capsular opacification and retrolenticular membranes. Proliferation of lens epithelial cells occurs, in which the cells undergo myofibrotic transformation, using the scaffold of the anterior capsule, posterior capsule, and even the vitreous face, which can lead to irreversible amblyopia (23–25). Capturing the IOL optic through both the anterior and posterior capsulotomy openings allows fusion of the capsular leaflets. It has been demonstrated experimentally that opposed capsules fuse together (26), and this fused apposition prevents migration of lens epithelial cells and prevents capsular opacification and retrolenticular membranes (13, 14, 22, 27–29). This fusion and fibrosis of the capsule (26) also contributes to the long-term support of the IOL and low probability of losing its captured position even after 20 years of follow-up (20).

The technique of double optic capture can be performed in the same surgical manner for adults and has been demonstrated to be a useful technique not only to secure primary and secondary posterior chamber IOLs but also to prevent the common occurrence of posterior capsular opacity in the adult population. Campos, Ahmed, and Shah presented a case of intraocular lens exchange of a sulcus placed single piece acrylic IOL which was causing uveitis, glaucoma, and hyphema (UGH) syndrome. After removal of the single-piece IOL, a three-piece IOL was placed in the sulcus and the optic was captured through both the anterior and posterior capsulotomy openings (30, 31). This demonstrated use of double optic capture to treat an inflammatory, acute uveitis condition confirms the ability of this technique to stabilize the IOL and prevent uveal chaffing even with placement of haptics within the ciliary sulcus. The authors state that double optic capture reduces IOL movement and ensures adequate centration (30).

The technique of double optic capture, as well as posterior capsulorhexis with optic capture, as a method to open the posterior capsule during the primary surgical procedure has also been utilized in adults to prevent posterior capsular opacity (PCO) following cataract surgery. Simply relying on the sharp edge design of IOLs is limited in effectiveness to prevent PCO and may be associated with symptomatic negative dysphotopsias (15). Nd : Yag (YAG) capsulotomy to treat visually significant posterior capsule opacity is not without risk, especially with regard to a higher incidence of retinal breaks or detachments (32). A meta-analysis study to evaluate the impact of YAG laser capsulotomy on the incidence of pseudophakic retinal detachment (RD) showed a relative risk ratio of 1.57 (33). Other complications of YAG capsulotomy include cystoid macular edema, elevated intraocular pressure, and damage to the intraocular lens (34). In addition, the cost of YAG capsulotomies is significant; in 2010, the Center for Medicare Services (CMS) spent 187 million dollars for YAG capsulotomies in the CMS population alone (35). Rupert Menapace in 2006 first described a technique of performing a primary posterior capsulorhexis after injecting ophthalmic viscosurgical device (OVD) under the posterior capsule opening to separate the underlying hyaloid surface from the posterior capsule (36). Arbisser in 2021 explains in detail the surgical method to perform hyaloid sparing PCCC using cohesive OVD to separate the capsule and anterior hyaloid with preservation of Berger’s space (37).

Menapace called this technique posterior optic buttonholing and reported no significant opacification within the visual axis in 500 consecutive patients (36). In 2008, he reported a series of 1,000 consecutive cases of this technique of posterior capsulorhexis with buttonholing and reported no cases of retrolental after-cataract formation (15). This technique would eliminate the need for sharp edge IOLs, thus decreasing the amount of associated visual distortion (15, 38).

Arbisser in 2022 describes that the hyaloid sparing technique can also be used with double optic capture (IOL optic being captured through both the anterior and posterior capsulotomies) (37). Arbisser describes this technique as hyaloid-sparing double capture with placement of the IOL optic into Berger’s space (37, 39). Dr. Thomas Oetting reported 12 cases from the University of Iowa for which his preferred method was this hyaloid sparing double optic capture resulting in IOL centration and no posterior capsular opacification (7).

## Conclusion

The double optic capture technique offers the advantage of 360° fusion of the capsules and less concern about posterior dislocation of an IOL due to too large or eccentric PCCC, and less concern about an IOL haptic becoming misplaced beneath the posterior capsulorhexis opening during the insertion process. In addition, the DOC technique allows for simultaneous posterior capsulotomy and anterior vitrectomy, which in the case of PHPV is necessary to remove any retrolental membranes or vitreous stalks while preventing secondary membranes and allowing long-term IOL centration. The technique of double optic capture has been shown to achieve IOL centration and near elimination of secondary cataract in infants, children, and even adults. Techniques involving double optic capture offer the future possibility of elimination of posterior capsule opacification in routine cataract surgery. In addition, future prospective randomized multicenter studies are in progress to evaluate long-term IOL stability in phacodenesis associated with pseudoexfoliation and long-term incidence of PCO in adults. Perhaps in the future, we will be able to utilize artificial intelligence solutions to evaluate the long-term stability of DOC and long-term biomechanical properties and centration, and finally develop models to create IOL designs to enhance the capturing of IOL optics.
